# Avian Satellite Cell Plasticity

**DOI:** 10.3390/ani10081322

**Published:** 2020-07-31

**Authors:** Maurycy Jankowski, Paul Mozdziak, James Petitte, Magdalena Kulus, Bartosz Kempisty

**Affiliations:** 1 Department of Anatomy, Poznan University of Medical Sciences, 60-781 Poznań, Poland; mjankowski@ump.edu.pl; 2 Prestage Department of Poultry Science, North Carolina State University, Raleigh, NC 27695, USA; pemozdzi@ncsu.edu (P.M.); jnppo@ncsu.edu (J.P.); 3 Department of Veterinary Surgery, Institute of Veterinary Medicine, Nicolaus Copernicus University in Toruń, 87-100 Toruń, Poland; magdalena.kulus@umk.pl; 4 Department of Histology and Embryology, Poznan University of Medical Sciences, 60-781 Poznań, Poland; 5 Department of Obstetrics and Gynaecology, University Hospital and Masaryk University, 601 77 Brno, Czech Republic

**Keywords:** satellite cells, plasticity, stem cells, chicken, embryo

## Abstract

**Simple Summary:**

Adult muscle regeneration and reconstruction is dependent on a population of adult stem cells, known as satellite cells. These cells were suggested to exhibit a certain degree of plasticity, being able to differentiate into lineages unassociated with muscle cells. In this study, we have used a range of visualization methods, as well as PCR, to identify a population of satellite cells obtained from samples of chicken muscles. Then, the cells, expressing a previously introduced detectable transgene, were introduced into chicken embryos and detected after three and eighteen days of their development. The traces of cell populations derived from the introduced satellite cells were detected in a range of embryonic tissues in both of the studied timeframes. The results of this study give further proof of the plasticity of muscle satellite cells, showing the potential locations of their migration during embryonic development.

**Abstract:**

Adult myogenesis is dependent on a population of precursor cells, located between the sarcolemma and the basal lamina of the muscle fiber. These satellite cells, usually present in a quiescent state, become activated in response to mechanical muscle strain, differentiating and fusing to add new nuclei to enlarging muscles. As their myogenic lineage commitment is induced on demand, muscle satellite cells exhibit a certain amount of plasticity, possibly being able to be directed to differentiate into non-myogenic fates. In this study, myosatellite cells were isolated from chicken muscle samples, characterized in vitro and introduced into developing blastoderms. They were further investigated using fluorescence microscopy, immunohistochemistry and PCR, to determine their location in embryos after three and eighteen days. The results of the in vitro analysis confirmed that the cells obtained from the *Pectoralis thoracicus* are highly myogenic, based on the expression of Pax7, Myogenin, MyoD, Desmin and the myotube assay. Furthermore, the investigation of satellite cells within the embryo showed their migration to the regions of *Pectoralis thoracicus*, heart, liver, gizzard, proventriculus, intestine and brain. Overall, the results of the study proved the high myogenicity of chicken *Pectoralis thoracicus* cell isolates, as well as provided new information about their migration pathways following introduction into the blastocyst. The presence of the introduced LacZ or eGFP transgenes across the embryo, even 20 days after myosatellite cell injection, further supports the notion that satellite cells exhibit significant plasticity, potentially transdifferentiating into non-muscle lineages.

## 1. Introduction

Adult myogenesis is dependent on a population of precursor cells, located between the sarcolemma and the basal lamina of the muscle fiber [1]. These satellite cells, usually present in a quiescent state, become activated in response to mechanical muscle strain, differentiating and fusing to add new nuclei to enlarging muscles [2]. This function implicates them in various processes of both normal and abnormal muscle function, as well as a range of pathological muscle-associated conditions [3].

Since their discovery, myosatellite cells have been classified to the pool of adult stem cells, with their differentiation restricted to the myogenic lineage [4]. However, recent studies of adult progenitors suggest that, through the process of transdifferentiation, their multipotency can be expanded into additional directions unassociated with their usual environment [5]. Satellite cells have been described as a heterogenous population, with their distinct sub-populations differing in activation kinetics, lineage marker expression and self-renewal timeframes. Approximately 80% of satellite cells are fused with myofiber, which allows them to react to muscle injury. The second population (~20%) was observed to remain predominantly quiescent, suggesting that they serve as a reserve, facilitating repair after significant muscle damage or strain [6]. Hence, as their myogenic lineage commitment is induced on demand, muscle satellite cells exhibit a certain amount of plasticity, possibly being able to be directed to differentiate into non-myogenic fates [7,8].

Despite those reports, the plasticity of satellite cells in the chicken model has not been extensively investigated. The limited study of chick satellite cells is surprising as the accessibility of the chick embryo makes it a suitable system to study the fate of cells injected into the blastoderm with relative ease [9,10]. It is also possible, using a surrogate eggshell culture system, to access the chick blastoderm and analyze the proliferation, migration and differentiation of cells injected at early embryonic stages [11].

The aim of this study was to evaluate the in vitro myogenic profile of freshly isolated chicken *Pectoralis thoracicus* cells, to confirm this muscle as a viable source of satellite cells. Furthermore, the cells were injected into blastocysts to examine the patterns of their migration in the embryo. The results should help to elucidate the extent of satellite cell plasticity, investigating their ability to incorporate into regions of the embryo unassociated with the muscle lineage.

## 2. Materials and Methods

### 2.1. Ethical Approval

All procedures involving animals were approved by the North Carolina State University Institutional Animal Care and Use Committee (19-717-B IACUC approved protocol 10/17/2019).

### 2.2. Satellite Cell Isolation

In all cases, the *Pectoralis thoracicus* from transgenic chickens expressing bacterial beta galactosidase [12] or green fluorescence protein [13] of varying ages, as indicated in the results, was employed as donor muscle, with the procedures patterned after the 1996 study of Mozdziak et al. [14]. Two hours before they were killed, to mark proliferating cells, the chickens were injected with 100 micrograms of 5-bromo-2′-deoxyuriine (BrdU) per gram of bodyweight. The extracted muscle, free of fat and any overlying connective tissue, was rinsed and placed in Hanks Balanced Salt Solution (HBSS). Subsequently, to enable satellite cell separation, each muscle sample was mechanically disassociated using sterile forceps and then incubated for 35 min in warm (37 °C) solution of 0.17% trypsin and 0.085% collagenase in HBSS (pH 7.4). Following enzymatic digestion, the tissue was washed twice with medium consisting of 84% DMEM, 15% Fetal Bovine Serum and 1% penicillin-streptomycin (all of the above were sourced from Sigma-Aldrich, Saint Louis, MO, USA). The tissue was resuspended in medium and triturated first through a Pasteur pipette and then with an 18-gauge needle. The cell concentration in the final suspension was estimated using a hemocytometer, with satellite cells either resuspended for embryonic injection or plated on 2-well chamber slides coated with 0.1% gelatin for in vitro evaluation.

### 2.3. Satellite Cell Immunocytochemistry

The isolated cells were maintained in in vitro cultures for varying periods of time, depending on the type of staining intended. Cells stained with Pax7, MyoD, myogenin or BrdU were left to attach to the cell culture wells overnight, while those stained with desmin were cultured for three days before fixation.

The cells destined for Pax7 staining were fixed with 4% paraformaldehyde for 30 min. Furthermore, cultures destined for MyoD, myogenin or desmin detection were fixed with cold methanol for 10 min, while those prepared for anti-BrdU staining were fixed for 10 min with 70% ethanol and denatured for 2 min using 0.07 NaOH to reveal the BrdU antigenic sites, with the NaOH subsequently neutralized with PBS.

In the next step, the cell cultures were incubated with their respective primary antibodies overnight at 4 °C. In all cases, parallel culture chambers were incubated without a primary antibody as a negative control. The Pax7 (Developmental Studies Hybridoma Bank, Iowa City, IA, USA) was diluted 1:1 with PBS 0.5% Tween + 10% Goat Serum (PBSTGS). The myogenin antibody (FD5; Developmental Studies Hybridoma Bank, Iowa City, IA, USA) was used as cell culture supernatant. Both Pax7 and myogenin were detected with Goat Antimouse IgG conjugated to FITC (Jackson Immunoreagents, Ely, UK) diluted 1:40 with PBSTGS. MyoD was detected with a rabbit polyclonal MyoD antibody (M-318, Santa Cruz Biotechnology, Dallas, TX, USA) diluted 1:40 with PBSTGS and detected with Goat Anti-Rabbit IgG conjugated to FITC (Jackson Immunoreagents, Ely, UK) diluted 1:50 with PBSTGS. The Desmin antibody (Developmental Studies Hybridoma Bank, Iowa City, IA, USA) was used as cell culture supernatant and was detected with Goat Anti–Mouse IgG conjugated to Horseradish peroxidase (Biomedia Holdings, Singapore) in conjunction with 3,3′-Diaminobenzidine (SIGMAFAST™, Sigma-Aldrich, MO, USA).

Finally, the cells subjected to myotube evaluation were cultured until they reached full confluency. Subsequently, the growth medium was removed and replaced with DMEM containing 2% FBS. The differentiation medium was changed every 24 h, the cells were maintained in such culture conditions for four days. The myotube cultures were fixed in 70% ethanol and stained with propidium iodide. Satellite cell differentiation was evaluated by determining the number of nuclei within myotubes. The total and fused nuclei numbers were determined to calculate a fusion index expressed as the number of nuclei lying within myotubes. The examination was performed following the procedures of Mozdziak et al. [15]. Five culture well replicates with ten observations per well were evaluated. A myotube was defined as containing 3 or more nuclei. The number of nuclei lying within myotubes was expressed relative to total nuclei.

### 2.4. Embryonic Injection

Freshly laid eggs were collected and checked for the presence of the embryo. Fertilized eggs were placed in surrogate chicken eggshells, prepared previously to allow embryo observation through a “window” cut out in the shell, covered by cling film, following the procedures of Borwornpinyo et al. [11]. A total of 10,000 freshly isolated satellite cells were injected into the subgerminal cavity using a pulled micro-pipette (50–60 micron-beveled, Humagen, Charlottesville, VA, USA). Subsequently, the chick embryos were cultured for three days, and they were then transferred into surrogate turkey eggshells and further incubated for up to 18 days. Subsequently, the embryos were collected for PCR analysis of the fate of the transgene, as well as for the immunocytochemical detection of cell fate.

The satellite cells were also labeled with PKH26 (a lipophilic cell membrane dye that is commonly used in in vivo cell tracking studies and it has a reported half-life of well over 100 days), following the manufacturers recommendations (Sigma-Aldrich, Saint Louis, MO, USA), and injected into chick blastocysts to analyze the migration of the cells. In this part of the experiment, the embryos were harvested after 3 days of incubation, with the location of marked cells examined using a fluorescence microscope (Leica, Wetzlar, Germany).

### 2.5. PCR

DNA was harvested from tissue samples using the Easy DNA kit supplied by Invitrogen (Carlsbad, CA, USA). The presence of the *lacZ* or eGFP gene in the offspring was determined using polymerase chain reaction. Briefly, template DNA (250 ng for *lacZ* or 1 μg for eGFP detection) was subjected to PCR using Taq polymerase (Fisher Scientific, Pittsburgh, PA, USA) to amplify, using specific primers, a 588 bp fragment of *lacZ* (Forward primer: 5′-TTCTGTATGAACGGTCTGGTC-3′; Reverse primer: 5′-ACTTACGCCAATGTCGTTATC-3′), or to amplify a 344 bp fragment of eGFP (Forward primer: 5′-CCTGAAGTTCATCTGCACCA-3′; 5′-AGTTCACCTTGATGCCGTTCT-3′). The DNA was amplified in a thermocycler (PTC-200, MJ Research, Waltham, MA, USA) using 40 cycles of 95 °C for 30 s, 54 °C for 1 min and 72 °C for 1 min. Subsequently, the amplification products were fractionated through a 1.5% agarose gel to reveal the presence of the amplified fragments. Positive control reactions using DNA obtained from confirmed transgenic lines, and two types of negative control, based on water and DNA from chickens that do not harbor the transgenes, were performed with each PCR run.

### 2.6. Immunohistochemistry

Only eGFP chickens were used to generate donor satellite cells for immunohistochemical analysis. Therefore, after 18 days of incubation after blastoderm injection, embryonic tissues were fixed in 4% paraformaldehyde in PBS, dehydrated, cleared and embedded in paraffin. Subsequently, 10-micron thick sections were deparaffinized, and hydrated. The sections were subjected to enzymatic retrieval with alpha chymotrypsin (1 mg/mL pH 7.8) at 37 °C for 20 min. The slides were rinsed in PBS and incubated overnight at 4 °C with a rabbit polyclonal antibody against eGFP (Invitrogen, Carlsbad, CA, USA) diluted 1:200 with PBS, 0.5% Tween and 10% goat serum. Subsequently, the sections were incubated for 2 h with goat ant-rabbit IgG conjugated to biotin, diluted 1:500 with PBS 0.5% Tween and 10% goat serum. The secondary antibody was detected using a Vectastain kit in combination with diaminobenzidine (Vector Labs, Burlingame, CA, USA).

### 2.7. Statistical Analysis

All of the statistical analyses were performed using MS Excel 2019 (Microsoft, Redmond, WA, USA) and Statistica 13.1 (StatSoft Polska, Kraków, Poland). The results were considered statistically significant at *p* < 0.05, with all of the data included in the manuscript fulfilling these requirements unless stated otherwise in the description.

## 3. Results

The first goal of the study was to evaluate the myogenicity of the satellite cell cultures in vitro, as this property is characteristic for this cell population [16]. The animals were pulse-labeled with BrdU, with their cells isolated and allowed to attach to chamber slides where they were evaluated using various immunomarkers. The observed cells were counted, with the results obtained from muscles of young birds (1 day or 3 weeks old) presented in Table 1.

Firstly, most of the isolated cells expressed desmin. Furthermore, virtually all nuclei were detected within myotubes following the fusion assay. When it comes to other markers, MyoD was expressed in most of the analyzed cells, while Pax7 and myogenin were expressed by 17% and 14% of the examined cells, respectively.

As the cells were collected from muscles of birds of different ages, the numbers of Pax7-positive cells were also evaluated in 14-week-old hens, with the results presented in Table 2.

As can be seen, no significant difference was found in Pax7 expression between the investigated donor ages. Secondly, an age-related increase in the number of BrdU-positive cells could be observed.

**Figure 1:**
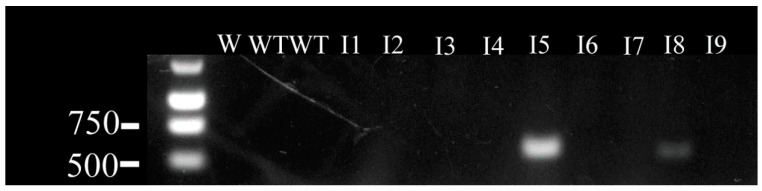
Presence of transgenes in embryos incubated for 3 days, evaluated based on the presence of a 588bp fragment of LacZ. W—water negative control, WT—wild type negative control, I—incubated embryo.

The next objective was to inject the cells into the chick blastoderm to examine their potential involvement in embryonic development. For this purpose, satellite cells were isolated from 1-year-old transgenic chickens carrying the lacZ gene and injected into the blastoderm of unincubated eggs. Subsequently, after three days, host embryos were evaluated for the presence of the gene using PCR. The sample results of gel electrophoresis aiming to detect the presence of the transgene in incubated embryos are presented in Figure 1.

The experiment was repeated three times, with every subsequent approach resulting in an improved number of LacZ+ embryos (Table 3).

The transgene was detected in 20–65% of the embryos, depending on the experimental repeat.

**Figure 2:**
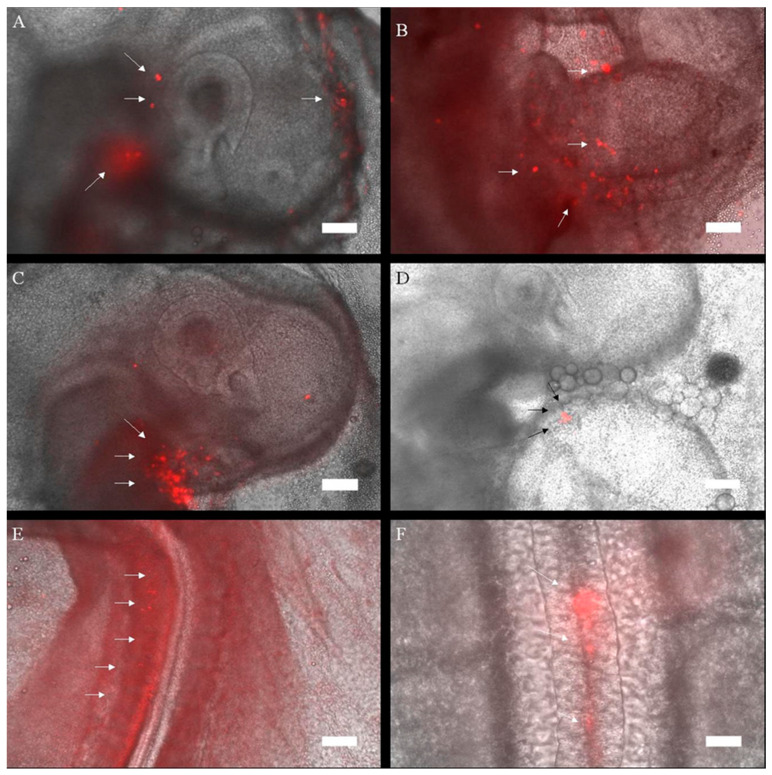
Composite pictures (20×) of LacZ cells donated from a Leghorn hen (60 weeks of age), stained with PKH-26, that were introduced into the embryo. All pictures were visualized under a fluorescent microscope within 64 h of incubation. (**A**) Head, (**B**–**D**) heart, (**E**) neural tube (upper section), (**F**) neural tube (middle section). Arrows indicate the PKH-26-stained cells.

Furthermore, the cells from 1-year-old laying hens were labeled with PKH26 (enabling stronger fluorescent labeling detectable in short-term embryonic development), with their fate evaluated after three days of incubation in the embryo. The results can be seen on the fluorescence microscopy photographs presented in Figure 2.

The cells appeared to specifically aggregate in three regions of the embryo. As can be seen on the pictures, as of three days of culture, the head, the heart and the neural tube were the endpoints of satellite cell migration.

**Figure 3:**
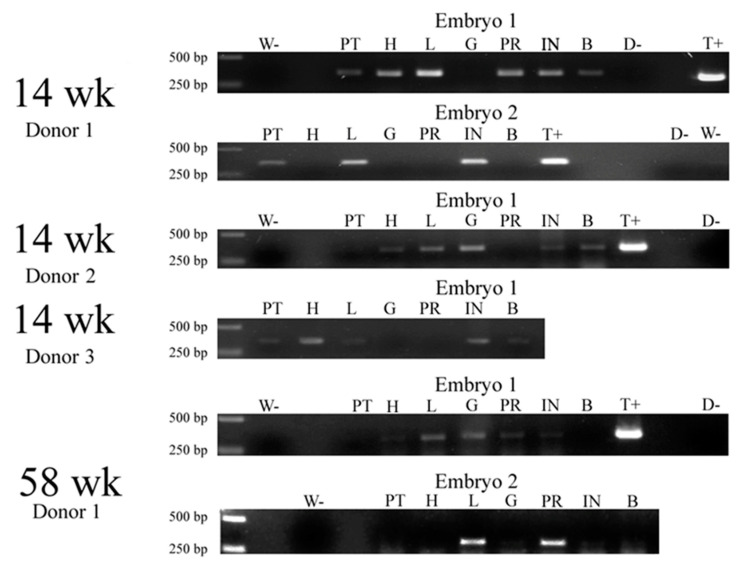
Presence of the transgene in embryos incubated for 20 days, evaluated based on the presence of a 344 bp fragment of eGFP. PT—pectoralis muscle, H—heart, L—liver, G—gizzard, PR—proventriculus, INT—intestine, B—brain, D-—DNA-negative, W-—water-negative, T+—transgenic-positive, wk—week.

Subsequently, the cells isolated from 14- and 58-week-old chickens harboring the GFP gene were injected into the blastoderm of fertile eggs, with the embryos incubated for 20 days. After that time, they were harvested and the DNA was isolated from specific organs and subjected to PCR to identify the presence of the transgene. The results of the analysis were presented in the form of gel electrophoresis pictures (Figure 3) and in a table summarizing the findings (Table 4).

The transgene was identified in a range of tissues, shedding further light on the migration of satellite cells to different parts of the organism, as well as the long-term incorporation of the lineages formed from these cells into the host tissue. As can be seen in the figure and table above, the transgene was identified in the muscle of *Pectoralis thoracicus*, the heart, liver, intestine and brain, and with a less pronounced presence in the gizzard and proventriculus. Furthermore, the transgene seems to manifest more rarely in the embryo incubated for 20 days cells when the cells are isolated from older chickens (58 vs. 14 weeks old).

**Figure 4:**
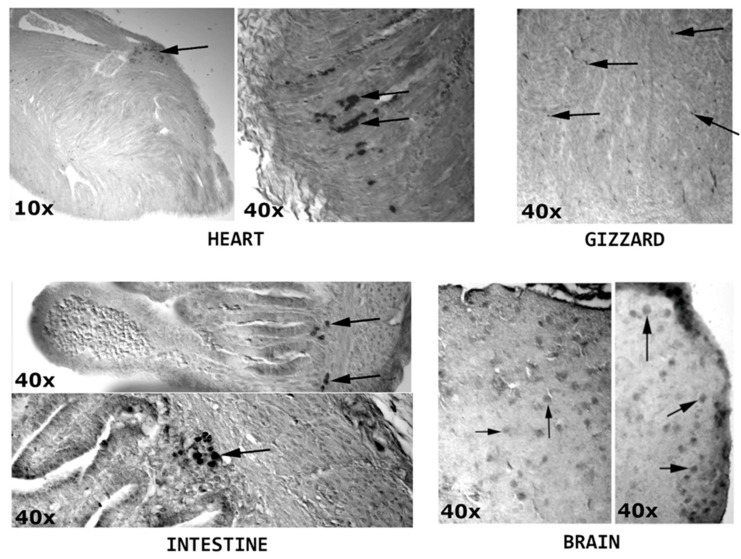
Microscopic images of the histological staining of embryonic tissues. Arrows indicate the eGFP-expressing cells of the injected satellite cell lineage.

Lastly, after the same injection and incubation procedure, the embryos were investigated using histochemistry, staining against eGFP. The results were documented on microscopic images, compiled in Figure 4.

As can be seen, aggregations of eGFP-expressing cells were observed in the heart, intestine, gizzard and brain. This stays in accordance with some of the PCR results, confirming the presence of lineages derived from satellite cells injected into the blastocyst after 20 days of embryo incubation, as well as several directions of injected myosatellite migration.

## 4. Discussion

Myogenic satellites are known to be the primary cells contributing to muscle growth and regeneration in vertebrates [17]. Furthermore, they consist of cells that only react in the need of muscle remodeling or reconstruction of significant scale [6]. These “dormant” myosatellites are thought to exhibit a certain amount of multipotency, some studies successfully differentiated them into lineages different than those making up the muscle tissue [18,19]. The accessibility of a chicken embryo made it a perfect platform to examine the in vivo plasticity of the myogenic satellite cells, due to the ease of observation during surrogate eggshell culture, the possibility to introduce cells directly into the blastocyst, as well as a large amount of experimental examples to serve as a reference in embryo visualization [20].

Hence, the first aim of the study was to obtain and confirm the identity of cells in chicken *Pectoralis thoracicus* muscle isolates from birds of different age. Pax7 expression was assessed for its specification as this marker is characteristic for satellite cells [21,22]. The levels of this protein were on average slightly lower in cells isolated from older chickens, however, this difference was not significant. These results could seem surprising, as the mitotic activity and viability of muscle stem cells was reported to gradually decline with age [23]. However, while there is a significant difference in age between the younger (1 day/3 weeks old) and older (15 weeks old) mature chickens examined in this part of the experiment, the latter group should not yet exhibit signs of aging-associated cellular senescence [24]. These results are also similar to the findings of a turkey model study by Doumit et al., in which satellite cells isolated from older specimens (15 vs. both 9 and 3 weeks of age) exhibited significantly higher mitotic activity in vitro [25].

Moreover, further myosatellite markers were examined in cells obtained from the 1-day-old chicks: MyoD and myogenin, as well as known myogenic regulatory factors and desmin, which is a generally accepted marker of satellite cells after three days in culture [19,26,27]. Desmin was detected in 100% of the isolated cells, while the levels of MyoD, myogenin and Pax7 reflected previously reported in vivo levels of cells expressing the three markers [28]. Finally, the ability of the isolated cells to fully differentiate into myotubes was evaluated during supplemented four-day culture, with around 99% of cells successfully assuming their terminal lineage, confirming their myosatellite identity [16].

Overall, the results suggest that the cells isolated from the chicken *Pectoralis thoracicus* are a highly myogenic cell isolate, independently of the age of the muscle donor, at least comparing between 1-day- and 15-week-old chickens.

Hence, in the next stage of the study, the cells obtained from LacZ-expressing chickens, allowing them to be later visualized [29], were injected into the chicken blastocyst, and detected after three days of incubation. Firstly, PCR was performed to detect the presence of the transgene in the incubated embryos. The experiment showed the presence of the donor cells in 20–65% of the resulting embryos three days after injection, proving their ability for survival and proliferation. The number of successfully obtained transgenic embryos increased with each attempt, possibly due to the optimization of the experimental workflow.

Furthermore, cells obtained from 60-week-old chickens were labeled with PKH26 allowing for their later detection (as this dye presents much better detectability than the LacZ or eGFP transgenes) [30], and injected into blastocysts in a procedure identical to the previous experiment. After three days of incubation, the cells were visualized in the resulting embryos to determine the location of introduced satellite cell migration. The cells were detected in different regions of the embryo, confirming their successful migration. They most prominently aggregated in the heart region, along the neural tube and in the head. This fact supports the notion that the satellite cells could exhibit some plasticity, with one possible explanation suggesting their possible transdifferentiation through a mesodermal precursor-like stage [31]. The locations in which the cells were detected could also support that theory, as mesodermal cells are the source of many of the heart structures [32], as well as can be found along the neural tube in the paraxial (somitic) [33] and head mesoderm, which gives rise to a range of muscles of the craniofacial region [34]. However, while this theory is present in the literature, it would need further experimental confirmation in the context of satellite cell plasticity.

In the final stage of the study, the satellite cell migration direction was examined after the 20-day incubation of the embryos after the injection of transgenic satellite cells expressing eGFP, with the use of PCR (based on DNA isolated from particular dissected embryonic organs), as well as immunohistochemistry. Using the former method, the transgene was detected in the heart, staying in accordance with the previous part of the study, the liver, along the gastrointestinal system (the proventriculus, gizzard and intestine), as well as in the brain. Immunohistochemical studies confirmed the location of transgenic cells in the heart, gizzard, intestine and brain. Overall, the results of the study further support the hypothesis of satellite cells plasticity, with the cells injected into the organism successfully incorporating into various tissues, allowing the introduced transgenes to be detected even after 20 days of embryo development. It is worth noting that the cells isolated from older chickens (58 vs. 14 week old) showed a notably lower rate of detection in the PCR study. This occurrence might be caused by the reported senescence of muscle progenitors associated with age [23]. Again, the mesodermal transdifferentiation theory is supported by the presence of the cells in the heart [32], as well as in the liver, the development of which involves significant participation of the mesodermal cells [35], and parts of the gastrointestinal tract, comprised of all three germ layers [36]. However, another explanation for transgenic cell presence needs to be found in the brain, primarily composed of cells of ectodermal origin [37]. A possible mesodermal connection could be based on the brain supplying blood vessels, the formation of which is dependent on the cephalic mesoderm [38]. However, the prominent presence of injected satellite cell-derived lineages in the brain could potentially be associated with their previously undescribed transdifferentiation abilities.

## 5. Conclusions

The traces of cell populations derived from the introduced satellite cells were detected in a range of embryonic tissues in both of the studied timeframes. Overall, the results of the study proved the high myogenicity of chicken *Pectoralis thoracicus* cell isolates, as well as provided new information about the pathways of their migration after introduction into the blastocyst. The presence of the transgene across the embryo, even 20 days after myosatellite injection, further supports the notion that satellite cells exhibit significant plasticity, transdifferentiating into non-muscle lineages, possibly through a mesodermal precursor-like stage.

## Figures and Tables

**Table 1:** In vitro myogenic profile of satellite cell isolates from young birds (1 day or 3 weeks old). The markers are sorted from those evaluated in cells isolated from early (top) to late stages of myogenesis (bottom) satellite cells. *n*—number of specimens examined.

Marker	*n*	Age of Donor	Weight of the Specimen (g) ± SD (Standard Deviation)	Counted Number of Nuclei	% Positive Cells/Nuclei
Early myogenesis					
BrdU	4	1 day	41 ± 4	35,953	1.3 ± 0.4
Pax7	4	1 day	41 ± 4	31,133	14 ± 4
Myogenin	4	1 day	41 ± 1	12,122	17 ± 6
MyoD	2	3 weeks	348 ± 80	384	74 ± 2
Desmin	4	1 day	39 ± 2	6473	100
Myotubes	2	1 day	39 ± 2	21,894	99 ± 0.4
Late myogenesis					

**Table 2:** In vitro myogenic profile of satellite cell isolates from mature hens (14 weeks old). *n*—number of specimen.

Marker	*n*	Weight of the Specimen (kg) ± SD	Cells Counted	% of Total Nuclei
BrdU	4	0.9 ± 0.03	6748	4.9 ± 1.7
Pax7	4	0.9 ± 0.03	4165	12.1 ± 2.1

**Table 3:** Survival of cells post-injection.

Repeat Number	Age of Muscle Donor	Number of Harvested Embryos	LacZ+ Embryos	% of LacZ+ Embryos
1	1 year	9	2	22
2	1 year	6	3	50
3	1 year	14	9	65

**Table 4:** The results of PCR analysis, with the number of embryos with positive transgene identification in certain anatomical regions indicated (PT—pectoralis muscle, H—heart, L—liver, G—gizzard, PR—proventriculus, INT—intestine, B—brain).

Age of Donors (Weeks)	Number of Donors	Total Number of Embryos	PT	H	L	G	PR	INT	B
14	3	4	3	3	4	1	1	4	3
58	2	2	0	1	2	0	2	1	0
